# Reducing Vaccinia virus transmission indoors within 60 seconds: Applying SAFEAIR-X aerosol with Iodine-V as a disinfectant

**DOI:** 10.1371/journal.pone.0279027

**Published:** 2023-01-27

**Authors:** Zoltán Köntös

**Affiliations:** IOl Investment Zrt, Budapest, Hungary; University of Rochester School of Medicine and Dentistry, UNITED STATES

## Abstract

Iodine-V ((C_26_H_39_N_4_O_15_)_x_ * (I_2_)_y_) demonstrates an *in vitro* virucidal activity by deactivating SARS-CoV-2 viral titers. It combines elemental iodine (I_2_) and fulvic acid (C_14_H_12_O_8_), forming a clathrate compound. The antiviral properties of Iodine-V reduce viral load in the air to inhibit viral transmission indoors. This antiviral property was applied to form a disinfectant solution called SAFEAIR-X Aerosol. The current study evaluates the antiviral efficacy of Iodine-V in aerosol form in a prototype called SAFEAIR-X Aerosol. The experiment measured the antiviral efficacy of SAFEAIR-X following exposure to the Vaccinia virus (VACV) samples as a confirmed surrogate for SARS-CoV-2. The SAFEAIR-X showed 96% effectiveness, with 2 seconds of spraying duration and 60 seconds of contact time releasing less than 0.0001 ppm of iodine into the air, and a log reduction value of 1.50 at 60 seconds in 2 out of 3 tests was observed. Therefore, this study demonstrates SAFEAIR-X aerosol as a potential indoor surface and air disinfectant.

## Introduction

The novel coronavirus known as severe acute respiratory syndrome coronavirus 2 (SARS-CoV-2) was first detected in December 2019 in Wuhan, China which manifests severe symptoms like difficulty in breathing and often results in the death of the affected person [1]. The route of transmission is respiratory fluids through which people are exposed to SARS-CoV-2 spread by an infected person [2, 3]. The small respiratory droplets and aerosol particles remain in the air for prolonged periods as they form dried fine droplets [4]. In contrast, larger virus-containing droplets condense and fall off quickly onto surfaces as compared to the smaller droplets, which remain in the air for an extended period [5, 6]. Scientific reports estimate that when a person coughs, 3,000 droplets are released, while a person’s sneeze releases over 40,000 droplets [7]. Hence, infected individuals risk spreading the virus into the air [8]. Therefore, health policy advisors guide the state government in maintaining social distancing based on this knowledge.

The viral load in the air gradually reduces because the heavier and larger respiratory droplets fall to the ground due to gravity, and the very fine droplets mix with the airstream [7, 9]. In addition, the viral load in the air also diminishes through the reduction of viral infections and viability over time due to environmental factors like humidity, temperature, and ultraviolet radiation [10–12].

SARS-CoV-2 is transmitted person-to-person *via* respiratory droplets and condensed viruses that exist for some time on surfaces [13, 14]. However, using disinfection methods in emergency departments, intensive care units, ambulatory clinics, and waiting rooms can significantly prevent the spread of the virus from infected asymptomatic individuals who might be transmission carriers in specific public settings. The virus could survive for several days on environmental surfaces, specifically in toilets, and still could be transmitted [15, 16]. It can remain viable on inanimate surfaces like glass, plastic, or metal for up to 9 days, but surface disinfection with ethanol (62–71%), hydrogen peroxide (0.5%), or sodium hypochlorite (0.1%) can adequately inactivate within a minute [17, 18].

The primary mechanisms for disseminating the SARS-CoV-2 virus are close physical contact and the shedding of viral particles from mucous membranes [19].

An ideal strategy for preventing viral transmission is decreasing the respiratory droplets in the air, in addition to the mechanical or natural ventilation that also reduces the viral load of SARS-CoV-2 [20]. Two air disinfection methods are upper-room germicidal ultraviolet fixtures and indoor air purifiers. Adequate ventilation with 6 to 12 room air changes per hour is recommended for air disinfection.

The current study applies Iodine-V as an aerosol called SAFEAIR-X, which is a prototype of air and surface disinfectant, to evaluate its efficacy against airborne viruses. Iodine-V (formula (C_26_H_39_N_4_O_15_)_x_ * (I_2_)_y_) is an aqueous solution that is composed of water-soluble elemental iodine (I_2_) and fulvic acid (C_14_H_12_O_8_) to form a clathrate compound. It forms a stable complex inert to nitrogen and oxygen in the air [20]. In addition, Iodine-V is safe, and an aqueous solution of 200 μg of iodine/mL is known as Essential Iodine Drops (EIDs); whereas the content of 20 μg of iodine/mL is called Incarvexx Essential Nasal Spray (Incarvexx) [20]. EIDs are an oral dietary supplement, and Incarvexx is recognized as a medical device. Recent studies [20] have shown Iodine-V’s efficacy against SARS-CoV-2. The antiviral activity of EIDs against SARS-CoV-2 was analyzed *in vitro*, and 99% efficacy was observed against SARS-CoV-2 in 3 dilutions [20]. The iodine drops were supplied through IOI Investment Zrt, which had a concentration of 200 μg of elemental iodine/ml.

Elemental iodine (I_2_) has broad-spectrum antimicrobial activity, and povidone-iodine (PVP-I) has been tested and applied for decades as an ideal antiseptic [21]. In PVP-I, I_2_ forms a complex with the polymer PVP (synthetic carrier), which has no antimicrobial activity. But Iodine-V is composed of I_2_ and fulvic acid (C_14_H_12_O_8_), a synthetic carrier and novel antimicrobial molecule [22]. I_2_ in PVP-I is a potent microbicidal agent with 99.99% virucidal efficacy at a concentration of only 0.23%, regardless of the virus type. Mechanism of action involves iodine rapidly penetrating microorganisms and important oxidizing nucleotides, fatty acids, and proteins causing cell death [23, 24]. An oral-nasal spray was recently developed that applied PVP-I directly, forming a coating or protective layer over the nasal and oral mucosa [25]. The SARS-CoV-2 could not bind with the ACE-2 receptor, so the spray prevented viral transmission or entry into the body. It is effective in the prevention of COVID-19. Also, PVP-I can destroy SARS-CoV-2, reducing the transmission of SARS-CoV-2 between patients [25]. Iodine-V is a recently designed novel complex that showed similar antiviral properties to PVP-I in an *in vitro* study against SARS-CoV-2 [20]. In both the complexes of Iodine-V and PVP-I, elemental iodine is the active substance.

The present research tested SAFEAIR-X Aerosol against the Vaccinia virus (VACV) as a surrogate of SARS-CoV-2. The selection of surrogate viruses is crucial when creating a standardized test. High resistance to drying and disinfectants, uncomplicated viral multiplication in cell culture, availability in virus collections, and a low BSL are prerequisites for conventional surrogate viruses (BSL 1 and 2) [26]. The VACV was recognized as a surrogate virus for the European program against enveloped viruses in 2015 to collect information faster about advancing enveloped viruses like Ebola or Zika due to the high biosafety level or the unavailability of viral samples in laboratories [26]. The decision was based on *in vitro* studies comparing modified Vaccinia virus Ankara (MVA/VACV) with MERS-CoV, SARS-CoV-1, and EBOV using PVP-I, which showed VACV to be the most resilient virus [27, 28]. When tested for the virucidal efficacy of alcoholic formulations for disinfectants, VACV was found to be safe and an appropriate surrogate. The use of the SAFEAIR-X Aerosol is thus a vital aid in preventing the transmission of viruses, including SARS-CoV-2, due to its antiviral activity with a relatively low concentration of 0.0001 ppm. In this study, we examined its potential use during the current pandemic.

## Materials and methods

### Objectives

The objective was to develop an aerosol in a device for domestic, pharmaceutical, veterinary, and commercial indoors use.

### Methods

In this experiment, SAFEAIR-X was tested against VACV as a surrogate of SARS-CoV-2. When evaluating the virucidal efficacy of alcoholic formulations for disinfectants, VACV is found to be safe and an apt substitute [29–31]. The vaccinia virus VR‐1549 Elstree strain was utilized in this experiment with standard EN 17272:2020 for chemical disinfectants and antiseptics, and techniques of airborne room disinfection were applied to evaluate bactericidal, fungicidal, mycobactericidal, pesticidal, protozoal, sporicidal, and virucidal, activities (EN 17272). The current research was conducted at BluTest Laboratories in Glasgow.

The experimental control used was according to standard EN 17272. The research aimed to examine the processes involved in dispersing a chemical disinfectant in gaseous, steam, or aerosol form. The test compared the number of microorganisms that survived the applied air disinfection process and analyzed the reduction in test microbes detected on nonporous surfaces [32]. The microorganisms can be inoculated with various standard pathogen strains, and reduction time was calculated relating to the untreated inoculated microbes. Table 1 lists the test and neutralization methods for the experiment provided by BluTest Laboratories.

**Table 1:** Test method and neutralization.

Method	The viral suspension was poured from a specific distance away and dried on steel discs. The inoculated carriers were treated without manual application *via* airborne disinfection from a predetermined distance in a room of a predetermined size.
	Cytotoxicity control, interference control, and neutralization control were utilized to validate the assays, and controls were used throughout the treatment to validate virus recovery.
Neutralizer	Dilution‐neutralization/gel filtration; Eagle’s Minimal Essential Medium (MEM) + 5% v/v fetal bovine serum (FBS) at 4°C.

The experiment was conducted over 14 days, from August 12 to August 27, 2021, taking the Vaccinia virus and Vero cells as the experimental specimens. The experimental conditions are listed in Table 2. The device used for this experiment was SAFEAIR-X, with a 100.0% concentration of the tested product. SAFEAIR-X was stable and non-reactive with water, dust, or other inorganic particles in the air. The chemical constituents of SAFEAIR-X (CAN A 50/50) are 70% ethanol (solvent), propane-butane gas (propellant gas), and Iodine-V (active substance). The combustion properties of propane-butane gas make it a propellant gas. A ratio of 1:1 with propane-butane gas and Iodine-V solution was added in CAN A 50/50. A solution was prepared with 100 mg of Iodine-V dissolved in 250 mL of 70% ethanol solution to form a 50/50 product. The viral strain known as Vaccinia virus VR-1549 Elstree strain was used in the experiment for evaluating the results. A specific purification protocol was not used to generate the virus for the assay performed in this research. All strains were taken from recognized culture collections, and quality control was performed accordingly. The only approach employed for preparing the viral stock was low-speed centrifugation to remove the cell debris from the final stock. Low-speed centrifugation is not considered a form of purification by many, but this procedure is sufficient for generating infectious, viable viruses with required titers for a successful assay. Table 3 outlines the identified viral strain and its passage (P) used as the Vaccinia virus VR‐1549 Elstree strain (P 4) and the identified cell type and its passage (P) used as Vero cells (P 16).

**Table 2:** Experimental conditions.

Period of analysis	August 12 to August 27, 2021
Product diluent used	NA
Product test concentrations	100.0% only
Room size	44.2 m^3^ (height: 2.70 m, width: 3.00 m, length: 5.45 m)
Distance to emission point (from carriers)	3.0 m
Height of test carriers	1.0 m
Contact time	t = 1 min ± 10 s
Temperature applied	20°C ± 1°C
Interfering substance	NA
Incubation temperature	37°C ± 1°C + 5% CO_2_

**Table 3:** Experimental specimen(s).

Viral strain and passage (P)	Vaccinia virus VR‐1549 Elstree strain (P 4)
Cell type and passage (P)	Vero Cells (P 16)

The Vaccinia virus was obtained and isolated in the laboratory in this experiment. The viral suspension was dried on a stainless steel disc and placed away at a defined distance [33]. The inoculated carriers were kept in a predetermined room size of 44.2 m^3^ and were treated *via* airborne disinfection from a distance without any manual application. The experimental conditions include the height of the test carriers at 1.0 m, contact time (t) was 1 min ± 10 s, the applied test temperature was 20°C ± 1°C, and the incubated temperature was 37°C ± 1°C + 5% CO_2_. SAFEAIR-X in the room was emitted from a distance of 3.0 m and a height of 1.5 m, with a relative height of 0.5 m from the carriers. Cytotoxicity control, interference control, and neutralization control were used to validate the assays. The virus was neutralized by diluting in Eagle’s Minimal Essential Medium (MEM) with 5% v/v fetal bovine serum (FBS) at 4°C, followed by gel filtration. The discs were transferred to a tube containing 1 ml of MEM with glass beads and vortexed to remove any residual viral particles from the surfaces. This dilution was the first step of neutralization. Microspin gel filtration column was used to complete the process by taking a small portion of the above suspension.

The virucidal activity of SAFEAIR-X was compared to a control. The inoculation and emission were given for 2 seconds, and contact with the Vaccinia virus VR-1549 Elstree strain was for 60 seconds, including the time of diffusion through the air of the confined room. Inhalation of less than 0.1 ppm concentration of iodine does not irritate the mucous membranes, but inhaling more than 0.1 ppm of iodine is lethal [34]. SAFEAIR-X can have a volume of 500 ml. A 2 second spray released less than 20 μg of iodine into a 44.2 m^3^ room, which released less than 0.0001 ppm of iodine content; hence, SAFEAIR-X exposure to the individuals in the room was no more than 0.1 ppm, which was harmless.

The experiment was performed thrice, and the samples of viral suspension were labeled as R1, R2, and R3.

### Protocol summary

The experiment was set up for a basic virucidal efficacy test with three test replicates and a contact time of 60 seconds. Inoculation of the virus was done on a stainless-steel disc and allowed to dry. The carriers were placed from a defined distance away from the emission point and facing away from the emission direction. The carriers were exposed to the disinfectant by air diffusion without manual application. Neutralization was done with serial dilutions, and titered virus was transferred to 96‐well tissue culture plates to estimate the viability of the virus through the median tissue culture infectious dose-50 (TCID_50_). TCID_50_ assay describes the virus dilution amount that can induce cytopathic effects (CPE) in 50% of wells containing experimental inoculated cell culture after a defined period of time. A parallel experiment was also conducted with the Vaccinia virus ATCC VR‐1549/Vero cells. TCID_50_ was calculated by using the Kärber method [33]. For the experiment, the starting titer of the virus was 3.17E+7 PFU/ml (Lg TCID_50_ 6.00).

### Cytotoxicity control

The experiment measured the effects of neutralized disinfectant on the host cells used to propagate the virus for determination of the sensitivity of the assay. The ISO18184 test relies on cultured host cells to measure the infectious viruses; the more infected host cells we identify, the more virus was recovered from the test. It happens because the virus kills these host cells in a characteristic way (called cytopathic effect or CPE), so the more infectious virus recovered, the more host cells are killed. For the assay to work, the virus (and only the virus) is killing the host cells should be ascertained. In this control, we add the liquid media to the test sample, wait for 5 minutes, recover the media, and add it to the host cells. The virus has been removed, so if the host cells die, toxicity must be from the test sample. A result like this can invalidate the whole test. It should also be noted that this test only assesses the cytotoxicity toward cultured cells growing in media under specific lab conditions and is designed to support the antiviral test’s conclusions.

### Interference control

The viral endpoint titration was exposed to 3 different sub-lethal concentrations of neutralized disinfectant and measured its effect on the pathogenicity of the virus concerning the titer achieved for control cells. This was conducted to evaluate the susceptibility of host cells to infection when a sub-lethal disinfectant concentration is applied. A lower susceptibility of host cells to infection after being treated by the disinfectant could lead to an overestimation of the product’s efficiency as fewer cells were infected. A volume of the neutralized disinfectant and a volume of phosphate-buffered saline (PBS) was applied in parallel to the host cells. The virus was then inoculated into the host cells, and the pathogenicity difference was estimated.

### Disinfectant suppression control

The highest disinfectant concentration was applied to the virus, and the mixture was instantly removed and neutralized. The neutralized viral titer was measured to evaluate the neutralization procedure.

### Viral recovery control

Two discs were inoculated in the laboratory under similar environmental conditions as an experiment with ‘Recovery Control—Start’ and ‘End’ controls and were allowed to dry. The experiment recovered one disc at exposure time (t = 0 minutes), ‘Recovery Control—Start’ before the beginning of the test, and the other disc, ‘Recovery Control—End,’ was left at room temperature for exposing the virus for control of degradation over time and then recovered. The viral recovery control was dried on a disc and treated with sterile water. This was done for 15 minutes and then recovered. This served a similar purpose as the standard 15-minute contact time start/end recovery control. These results indicated a stable condition for the virus under experimental conditions and were not significantly degraded.

### Reference virus inactivation control

The virus was exposed to 0.7% W/V formaldehyde, and TCID50 assessed the viral recovery at 5 and 15 minutes to examine the retained reproducible disinfectant resistance when the test virus was added. In addition, the cytotoxicity of neutralized formaldehyde was measured to assess the sensitivity of the assay.

### Ethical approval and patient consent

The current study only used the Vaccinia virus VR‐1549 Elstree strain and excluded human participants. Hence, ethical approval and patient consent were not needed for this study.

### Statistical analysis

The viral titers were validated using the Kärber method and expressed as TCID_50_/ml with standard deviations [35, 36]. The difference between the viral titer following contact time with the aerosol and the control viral titer results in titer reduction [37, 38].

The research aimed to identify the viral efficacy of SAFEAIR‐X with a 2-second emission time and a 60-second contact time. Hence, no other theoretical analysis was done, and no more test data were recorded.

## Results

The effectiveness of SAFEAIR‐X as a disinfectant for indoor settings against VACV was observed. The SAFEAIR‐X Aerosol covered an enclosed area with fine dry droplets, and the device is stable in presence of water, dust, or other inorganic particles lingering in the air.

The prototype SAFEAIR-X Aerosol samples showed considerable virucidal efficacy against VACV. The contact time was only 60 seconds in the experiment for observation, but, as per the protocol, the measures of viral control were taken at different time intervals. Tables 4 and 5 show the results obtained from the SAFEAIR-X and control experiments.

**Table 4:** EN 17272 surface test for the efficacy of SAFEAIR‐X (CAN A 50/50) against Vaccinia virus ATCC VR‐1549 (20°C ± 1°C) with virus control.

Control									
Recovery Control		Recovery Control		Virus Recovery		Cytotoxicity		Disinfectant	
Start		End		15 min				Suppression VS	
raw data	TCID_50_/ml	raw data	TCID_50_/ml	raw data	TCID_50_/ml	raw data	TCID_50_/ml	raw data	TCID_50_/ml
4.00	3.16E+05	3.67	1.47E+05	3.50	1.00E+05	0.00	3.16E+01	4.33	6.81E+05
666600	3.16E+05	666400	1.47E+05	666210	1.00E+05	0.00	3.16E+01	666611	6.81E+05

**Table 5:** EN 17272 surface test for the efficacy of SAFEAIR‐X (CAN A 50/50) against Vaccinia virus ATCC VR‐1549 (20°C ± 1°C) with virus control.

Product	Interfering substance	Concentration	Lg TCID_50_		LRV^a^ Contact Time	Efficacy^b^
			0 min	1 min	1 min	(%)
SAFEAIR-X	n.a.	100.0% (v/v) R1	6.00	5.17	0.83	85.21
SAFEAIR-X	n.a.	100.0% (v/v) R2	6.00	4.50	1.50	96.84
SAFEAIR-X	n.a.	100.0% (v/v) R3	6.00	4.50	1.50	96.84
Virus control	Clean		5.50	5.17	0.33	n.a.
Formaldehyde	PBS	0.7% (w/v)	n.a.	n.a.	n.a.	n.a.

^a^ LRV (log reduction value) reduces the virus compared to the virus control. n.a. = not applicable

^b^ Efficacy: 100*(1–10^-LRV^)

According to the TCID_50_ formula, a value of ‘0.00’ represents ‘3.16E+01’which indicates a lower limit calculation instead of the presence of the virus. The number of wells (96-well plate) positive for a cytopathic effect (CPE) was noted and converted to a proportion of total wells. The cytotoxicity level was calculated using the formula 10*10 ^(n+0.5)^ [31]. The calculation for cytotoxicity control for values with no CPE recorded is 10*10 ^(n+0.5)^ = 31.6. The log of this value is 1.50. This defines the lower limit of the calculation.

The cytotoxicity of the prototype solution had no effect on cell morphology, growth, or susceptibility to the virus in experimented mixture dilutions. SAFEAIR-X product did not show any cytotoxicity.

This procedure’s validation of neutralization is known as the disinfectant suppression test. Column chromatography through an Illustra Microspin S‐400 HR column was performed to neutralize the disinfectant to achieve the best validation results. There was effective neutralization of the virucidal activity of the disinfectant at a dilution concentration of 100.0% v/v, as the difference observed was not greater than 0.5 log_10_ for the virus.

Table 5 compares the surface tests for SAFEAIR-X efficacy and virus control. The contact time of SAFEAIR-X had a contact time of only 60 seconds. However, as per the protocol, the virus recovery control was taken as a reference before and after the exposure time. R1 at 60 seconds shows an LRV of 0.83, while R2 and R3 have an LRV of 1.50, as mentioned in Table 5. Also, the efficacy at 60 seconds for R1 was observed to be 85%; for R2, it was 96%, and for R3, it was 96%. Hence, the benefits of SAFEAIR-X in inactivating VACV in a confined space were analyzed.

Table 6 shows the validation data for the interference control.

**Table 6:** Interference control.

Virus dilution						
Log of virus dilution	‐3	‐4	‐5	‐6	‐7	‐8
PBS Control	1	1	1	0.83	0.17	0
	3.16E+02	3.16E+02	3.16E+02	2.14E+02	4.68E+01	3.16E+01
	2.50	2.50	2.50	2.33	1.67	1.50
Raw Data^a^	6	6	6	5	1	0
Product	1	1	1	1	0.33	0
	3.16E+02	3.16E+02	3.16E+02	3.16E+02	6.76E+01	3.16E+01
	2.50	2.50	2.50	2.50	1.83	1.50
Raw Data^a^	6	6	6	6	2	0
Log Difference	0.00	0.00	0.00	‐0.17	‐0.16	0.00
Product Cyt Dilution	‐1	‐1	‐1	‐1	‐1	‐1
PBS Dilution	Neat	Neat	Neat	Neat	Neat	Neat

^a^ Raw data: log of stock virus (TCID_50_)

The disinfectant-treated and PBS-treated cells showed a difference of (-0.16 + -0.17) = -0.33 log_10_, which was less than 1.0 log_10_, suggesting no interference of cytopathic effects with the dilutions of the disinfectant to sub-acute levels of viral production. Table 6 confirms the validity of the test results.

## Discussion

Iodine has broad-spectrum antimicrobial activity against bacterial, viral, fungal, and protozoan pathogens and has been used as an antiseptic to prevent infection and treat wounds for decades. Coronavirus transmission can be primarily influenced by humidity, but the temperature could still moderately regulate the spread of COVID-19 [10]. Existing evidence supports an increased SARS-CoV-2 replication at upper airway temperatures (25–33°C when breathing) relative to temperatures in the lower airways (37°C) [11]. For SARS-CoV-2, the reproductive number (the count of secondary cases arising from a primary case in a susceptible population) is approximately 2 to 3, with estimates in some cases being as high as 5.7 [12].

Based on past research, viruses are also classified according to the increasing difficulty of inactivating the viruses *via* chemical disinfection. These are categorized as enveloped viruses, large unenveloped viruses, and small unenveloped viruses. SARS-CoV-2, monkeypox, and VACV are examples of enveloped viruses that are more sensitive to chemical disinfectants than their non-enveloped counterparts [39]. Iodine-V is a halogenated substance disinfectant used as an oxidizing agent [20]. Iodine-V, a potent virucidal drug that uses elemental iodine, demonstrated its effectiveness against SARS-CoV-2 by reducing the viral titer by 99% (LRV 2.0). Iodine is a strong oxidizing agent that destroys both small, non-enveloped viruses like norovirus and enveloped viruses like coronavirus or influenza [20]. Iodine-V is advantageous with better stability and improved potency *in vivo* and may be considered a nasal or oral antiseptic to reduce virus transmission [20].

Though the origins of the broad-spectrum antiviral activities of PVP-I have not been fully studied, there is evidence showing that iodine inhibits virus attachment to the host’s cell surface by blocking its receptors. It can also inhibit the enzymatic activity of viruses such as neuraminidase which are necessary to release viruses from host cells that prevent the infection spread to other uninfected cells [39]. Chemical agents more easily inactivate viruses in suspension than viruses in dried form on surfaces.

SAFEAIR-X Aerosol prototype is analyzed for its virucidal efficacy on the VACV cultures grown in Vero cells, where VACV was taken as a surrogate for the SARS-CoV-2 along with a control. It was tested on surfaces in dried form. SAFEAIR‐X was tested under standardized, nearly real-world settings to observe the effectiveness of inactivating viruses at the lowest concentration of 0.0001 ppm. The experiments supported the aerosol’s performance in a lab setting and highlighted SAFEAIR‐X’s potential to be applied as a disinfectant. The aerosol was exposed to the confined room for only 60 seconds. Viral titers were calculated as per the Kärber method, and the virucidal efficacy was measured as the median tissue culture infectious dose (TCID_50_). This assay was validated by cytotoxicity, interference, and neutralization controls. Table 4 illustrates the cytotoxicity data as 0.00 or 3.16E+01 (TCID_50_/ml), which indicates that there were no cytotoxic effects of the SAFEAIR-X aerosol on VACV in the diluted samples; the starting viral titer remained as such. Also, the viral morphology and growth remained unchanged, demonstrating negative cytotoxic effects. Table 4 also depicts the neutralization control validation with disinfection suppression control test done by column chromatography and shown to effectively neutralize the disinfectant’s virucidal activity at a concentration of 100%. Table 6 illustrates the interference control in which no significant difference was found between the disinfectant-treated and PBS-treated cells. This validates the assay by showing that the disinfectant at various dilutions did not interfere with the virus for exerting its cytopathic effects. The viral suspension exposed and dried for 15 minutes was checked by virus recovery to confirm the control of degradation on an exposed surface. The virus remained stable and did not degrade significantly (1.47E + 05 TCID_50_/ml at the Recovery Control “End” and 1.00E + 05 TCID_50_/ml for virus recovery).

The experiment gave reproducible results in three repeats which are demonstrated in Table 5 with SAFEAIR-X taken at 100.0% (v/v) concentration (R1, R2, and R3). There was a reduction in the viral titer in all three, with virucidal efficacy at 85.21%, 96.84%, and 96.84% for R1, R2, and R3, respectively. All these results confirm the efficacy of SAFEAIR-X Aerosol prototype to exert antiviral effects on the experimental VACV/Vero cells and validate the findings.

## Conclusion

The study aimed to confirm the use of Iodine-V, a component of air disinfectant for indoor settings [20]. The prototype SAFEAIR-X Aerosol was observed to be highly effective against VACV. The three replicable experiments demonstrated an average of 93% inactivation of viruses within 60 seconds in a 44.2 m^3^ confined room. SAFEAIR-X can significantly reduce the viral load on surfaces in indoor settings within 60 seconds and theoretically in the air because halogen gases are highly efficacious against viruses in lower concentrations [40, 41]. Iodine has a broad range of antimicrobial applications with efficacy against viruses. Hence, SAFEAIR-X Aerosol can effectively reduce the risk of indoor transmission of SARS-CoV-2 and other enveloped viruses, including the monkeypox virus, between humans and animals in indoor areas.

SAFEAIR-X is also safe; it releases only 0.0001 ppm of iodine into the room during the research, giving options for extending the spray time or increasing the aerosol concentration to enhance its efficacy further while still maintaining a safety limit under 0.1ppm.

## Abbreviations

SARS-CoV-2: severe acute respiratory syndrome coronavirus 2
COVID-19: Coronavirus disease 2019
VACV: Vaccinia virus
SAFEAIR: X: SAFEAIR-X Aerosol
EIDs: Essential Iodine Drops
Iodine-V: Iodine fulvic acid clathrate complex
PBS: Phosphate-buffered saline; Incarvexx: Incarvexx Essential Nasal Spray
PFU: Plaque Forming Unit
